# Personalized aesthetic management of skeletal Class II malocclusion with a combined approach of orthodontic, orthognathic, and prosthodontic treatment: A case report

**DOI:** 10.1097/MD.0000000000041326

**Published:** 2025-02-07

**Authors:** Jinhan Nie, Min Hu, Huichuan Qi, Zhi Mao, Zhina Wu, Mengna Duan, Guomin Wu, Xue Yang, Yi Zhang

**Affiliations:** a Department of Orthodontics, Hospital of Stomatology, Jilin University, Changchun, Jilin, P.R. China; b Jilin Provincial Clinical Medicine Research Center of Orthodontics, Changchun, Jilin, P.R. China; c Department of Prosthodontics, Hospital of Stomatology, Jilin University, Changchun, Jilin, P.R. China; d Plastic Aesthetic Center, Hospital of Stomatology, Jilin University, Changchun, Jilin, P.R. China.

**Keywords:** maxillary segmental retraction, orthodontic–orthognathic treatment, personalized treatment, 6 elements of orofacial harmony, skeletal Class II malocclusion

## Abstract

**Rationale:**

The 6 elements of orofacial harmony play a crucial role in establishing individual aesthetics in orthodontics and orthognathic treatments. The use of digital tools streamlines multidisciplinary treatment processes and facilitates the attainment of precise outcomes.

**Patient concerns:**

The patient required orthodontic treatment because of a convex profile. UR1 was missing because of trauma and was replaced with a fixed bridge.

**Diagnoses:**

The patient presented with a skeletal Class II relationship and a deep overbite. Diagnosis based on the 6 elements of orofacial harmony identified an overdeveloped maxilla, a relatively normal mandible, and an underdeveloped chin with a vertical growth pattern.

**Interventions:**

To address these issues, a combined orthodontic and orthognathic treatment plan was implemented with a detailed surgical protocol developed according to these 6 elements. Customized brackets and 3D-printed occlusal plates were used in this study. After completing orthodontic treatment, an anterior prosthesis was designed using 3D digital technology to restore occlusal function and enhance anterior aesthetics.

**Outcomes:**

Post-treatment, the patient demonstrated reduced facial convexity, a flattened nasolabial sulcus, balanced occlusal forces, and restored occlusal function.

**Lessons:**

The outcomes of this case highlight the crucial role of the 6 elements of orofacial harmony in guiding the design and aesthetic planning of orthodontic and orthognathic treatment. In addition, the use of personalized treatment tools significantly enhances the treatment accuracy.

## 1. Introduction

The 6 elements are aesthetic evaluation criteria derived from the analysis of coordinated maxillofacial populations.^[1]^ These elements integrate soft and hard tissue landmarks to define the standard range of the dental arch, maxilla, and mandible in three-dimensional space.^[1]^ For skeletal Class II patients who generally seek treatment to correct profile convexity,^[2]^ both simple tooth extraction and orthognathic surgery can effectively enhance the facial profile.^[3]^ However, the choice of treatment varies significantly depending on the deformity type. Therefore, accurate identification of the deformity type is essential for formulating an effective treatment plan, with the 6 elements playing a critical role in this process.^[1,4]^ By quantifying the deviation of teeth and craniofacial bones from normal ranges, the 6 elements assist in selecting appropriate treatment options and determining the target positions.^[1,5]^

Aesthetics and functionality are common goals in oral treatment, including orthodontic, orthognathic, and prosthodontic approaches. The widespread adoption of personalized treatment tools would facilitate the achievement of these goals. The use of customized brackets and 3D-printed occlusal plates can streamline the process and improve the accuracy of combined orthodontic–orthognathic treatment.^[6,7]^ For prosthetics, integrating facial scans, intraoral scans, digital face-bow transfers, and digital cross-articulations can generate a 3D virtual patient model.^[8]^ This model incorporates facial soft tissues into the design process and accurately simulates the intraoral conditions. Such integration significantly addresses both occlusal functionality and aesthetic requirements in restorative treatments.^[9]^

In this case, the principle of ensuring aesthetics and functionality was consistently upheld. These 6 elements served as a diagnostic guide to establish a treatment strategy for combined orthodontic–orthognathic treatment and formulate a tailored surgical plan. Personalized treatment tools such as brackets, 3D-printed occlusal plates, and 3D virtual patients have been used to improve treatment precision and accuracy.

## 2. Case presentation

### 2.1. Diagnose

**Figure 1.**
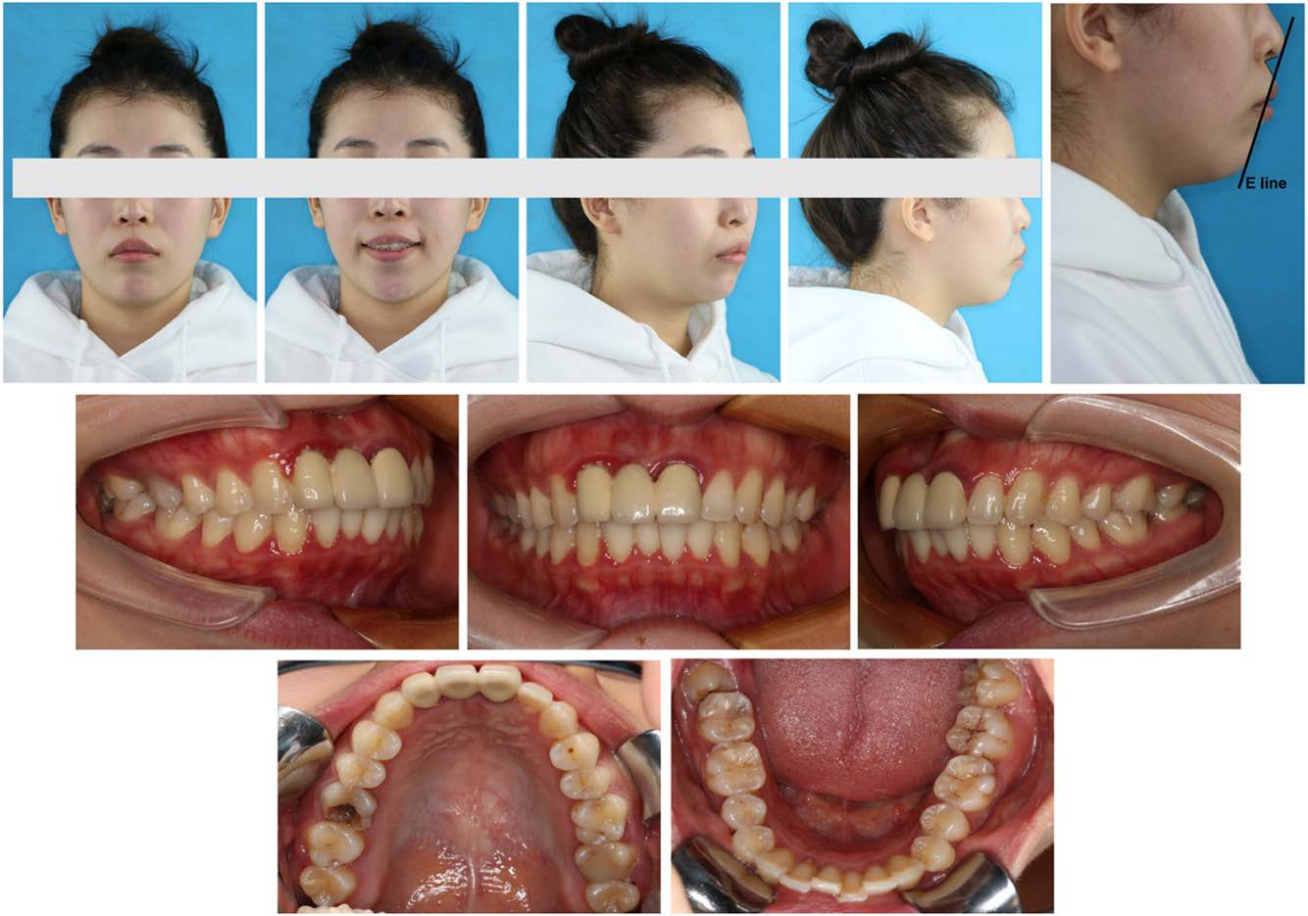
Pretreatment facial and intraoral photographs.

**Figure 2.**
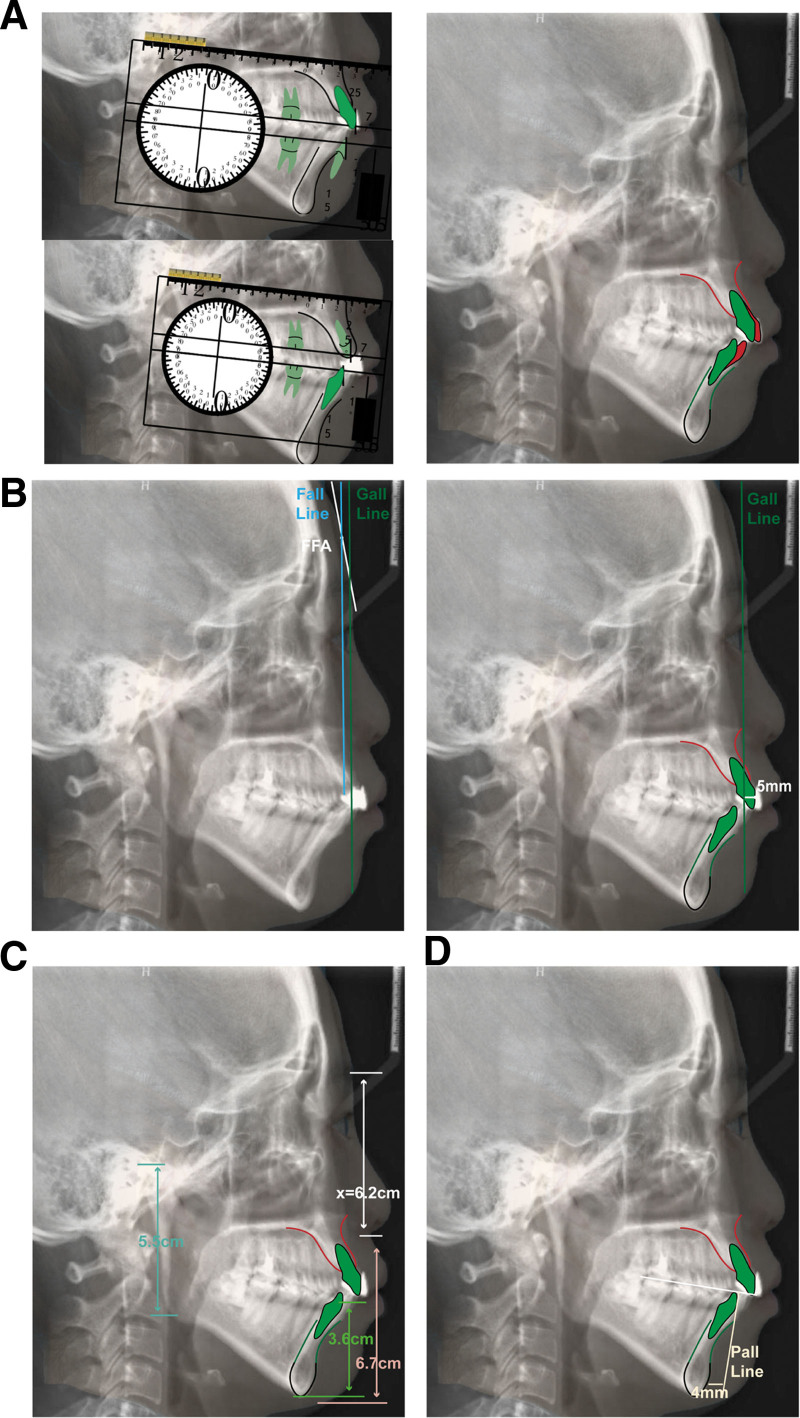
Andrews’ 6 elements analysis. (A) Element I, maxillary incisors were retracted by 2 mm, and mandibular incisors were retracted by 4 mm. (B) Element II, determine the GALL line, with the adjusted maxillary incisor FA point located 5 mm anterior to the GALL line. (C) Element IV, anterior subfacial height is slightly greater than the posterior height. (D) Element V, determine the PALL line, the pogonion is located 4 mm posterior to the PALL line.

**Figure 3.**
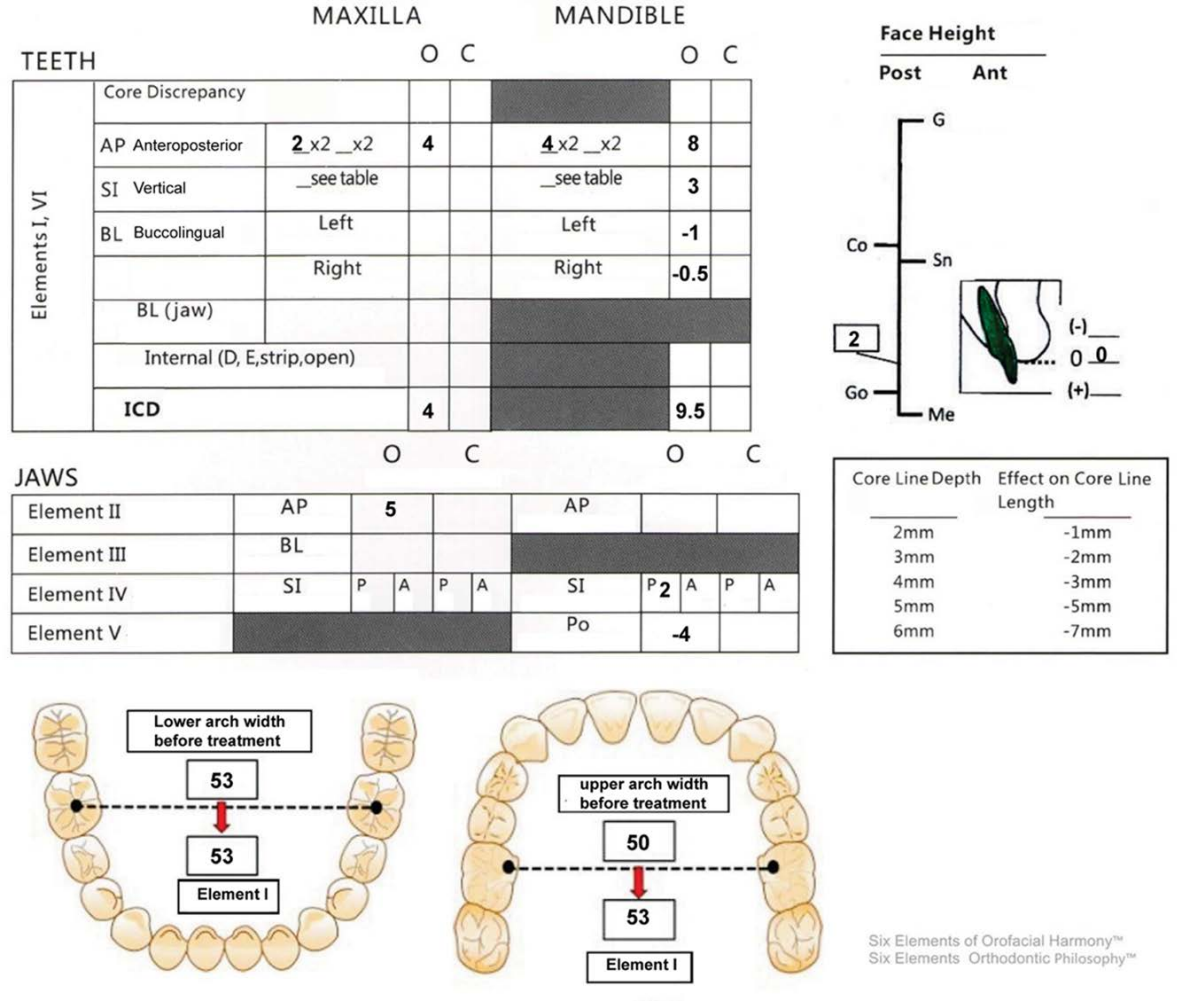
Diagnose in 6 elements.

**Figure 4.**
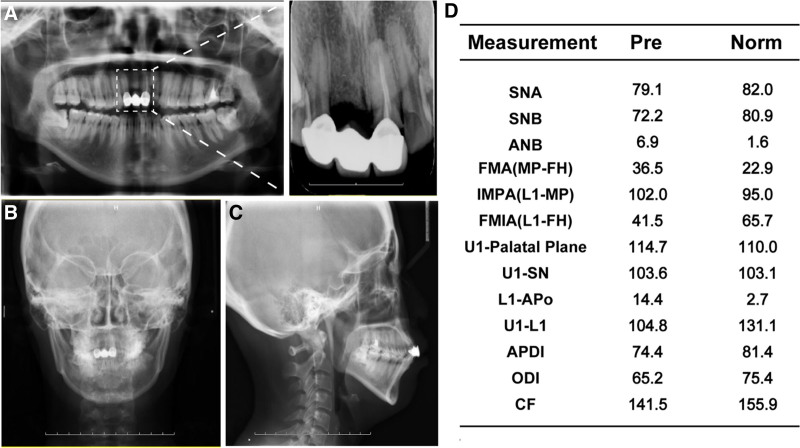
Pretreatment radiographs. (A) Lateral cephalogram. (B) Posteroanterior cephalogram. (C) Panoramic radiograph. (D) Cephalometric analysis.

A 24-year-old female sought orthodontic treatment for a convex mouth. A detailed diagnosis was conducted under the guidance of 6 elements (Table S1, Supplemental Digital Content, http://links.lww.com/MD/O298), focusing on the three-dimensional aspects of the soft, dental, and skeletal (Table 1, Figs. 1–4).

**Table 1:** Diagnostic table.

Sagittal	Vertical	Horizontal	Others
*Soft issue*			
Sharp nasolabial angle; Upper lip 2 mm anterior to E line; Lower lip 3 mm anterior to E line (Fig. 1).	3 mm underlip; visible gingiva (Fig. 1).	Levelled both side of mouth	
*Dental*			
5 mm overjet; Class I (Fig. 1). Upper incisors 2 mm labially inclined; Lower incisors 4 mm labially inclined (Fig. 2A, Fig. 3).	3 mm overbite; 4 mm Spees’ curve (Fig. 1, Fig. 3).	Narrow upper arch (Fig. 1, Fig. 3).	UR6 caries to pulp; UR1 (-); UL1 (RCT) (Fig. 4).
*Skeletal*			
Skeletal class II; Maxilla 5 mm protrusion; Mandible normal (Fig. 2B, Fig. 3). Chin 4 mm underdevelopment (Fig. 2D, Fig. 3).	Maxilla normal; anterior subfacial height greater than the posterior height (Fig. 2C, Fig. 3).		

RCT = root canal treatment.

### 2.2. Treatment alternatives

Several treatment options have been proposed to address patient concerns regarding facial convexity (Table 2).

**Table 2:** Treatment options.

Options	Extraction	Orthognathic	Prosthodontic	Advantages	Disadvantages
1	UL1, LL4, LR4, UR6	Maxillary LeFort I osteotomy; genioplasty	Anterior restoration	Remove affected teeth. aesthetics improvement.	Long preparation; high costs; significant damage.
2	UL4, UR4, LL4, LR4, UR6	Maxillary LeFort I osteotomy; genioplasty	UR1 implant.	Short preparation time; aesthetics improvement.	Tooth lifespan limited; high costs; significant damage.
3	UL4, UR4, LL4, LR4, UR6	Genioplasty	UR1 implant.	Low cost; minimal damage.	Aesthetics improvement poorly; tooth lifespan limited.

Considering the etiology of the patient’s skeletal protrusion and pursuit of aesthetics, combined maxillary orthognathic surgery is a superior choice compared to simple tooth movement. Additionally, compared with extracting healthy first premolars, removing the UL1 that underwent root canal treatment aligns better with the principle of prioritizing the extraction of diseased teeth. Therefore, option one has been determined as the treatment plan for this patient. Furthermore, due to the deep caries and prolonged duration associated with UR6, along with intra-pulpal absorption and significant treatment difficulty, long-term efficacy is poor. In contrast, both root and crown morphologies of UR8 were good. Hence, we chose to extract UR6 and sequentially shift the posterior teeth forward to compensate for this.

### 2.3. Treatment plan

**Figure 5.**
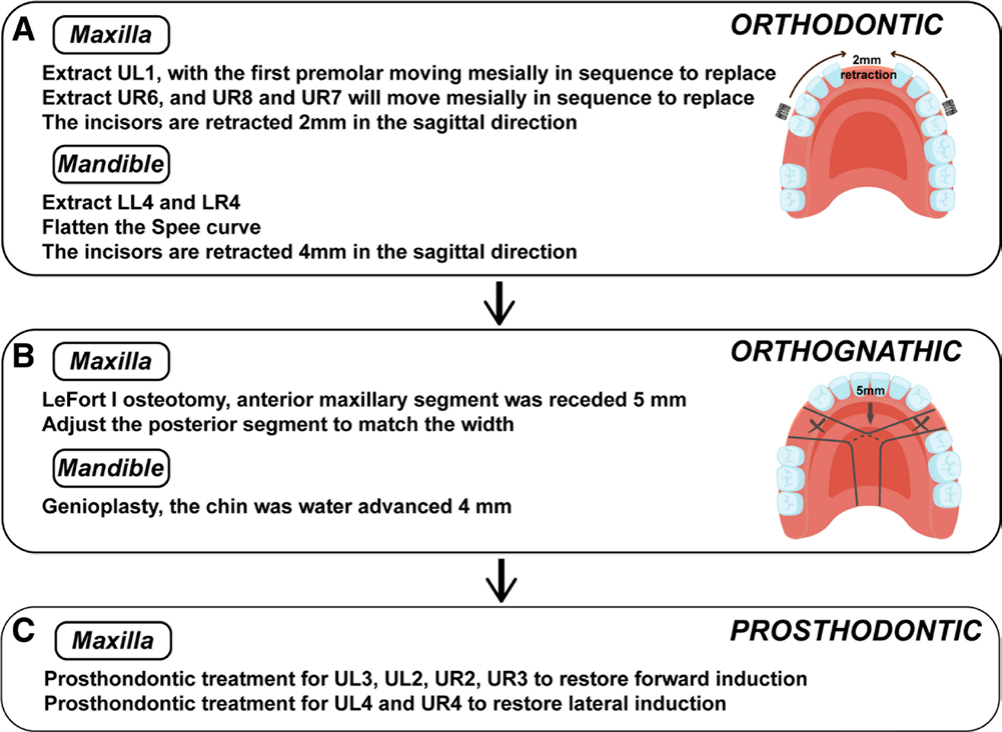
Diagram of treatment plan.

In the maxilla, the UL1 was extracted to centralize the gap between the premolars, while the incisors were retracted by 2 mm. Additionally, UR6 was extracted, and UR7 and UR8 were repositioned anteriorly (Fig. 5A). In the mandible, LL4 and LR4 were extracted, and the extraction gaps were used to flatten the curve, with the incisors retracted by 4 mm (Fig. 5A). A Lefort I block osteotomy was performed in the maxilla, retracting the anterior bone segment by 5 mm and adjusting the posterior bone segment to achieve coordinated width (Fig. 5B). A surgical procedure was performed in the mandible to advance the chin by 4 mm (Fig. 5B). Following orthodontic and orthognathic treatment, a maxillary anterior dental prosthesis was used to restore forward and lateral guidance (Fig. 5C).

### 2.4. Presurgical orthodontics

**Figure 6.**
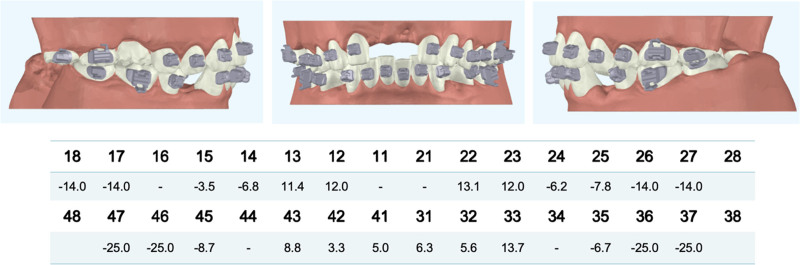
The data of customized bracket base plate.

**Figure 7.**
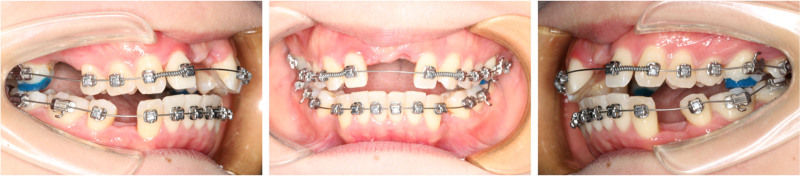
Bracket bonding intraoral photographs.

Once the target orthodontic position was determined, customized brackets were fabricated to simplify the orthodontic procedure by prefabricating the bracket base (Figs. 6 and 7).

### 2.5. Surgical options

**Figure 8.**
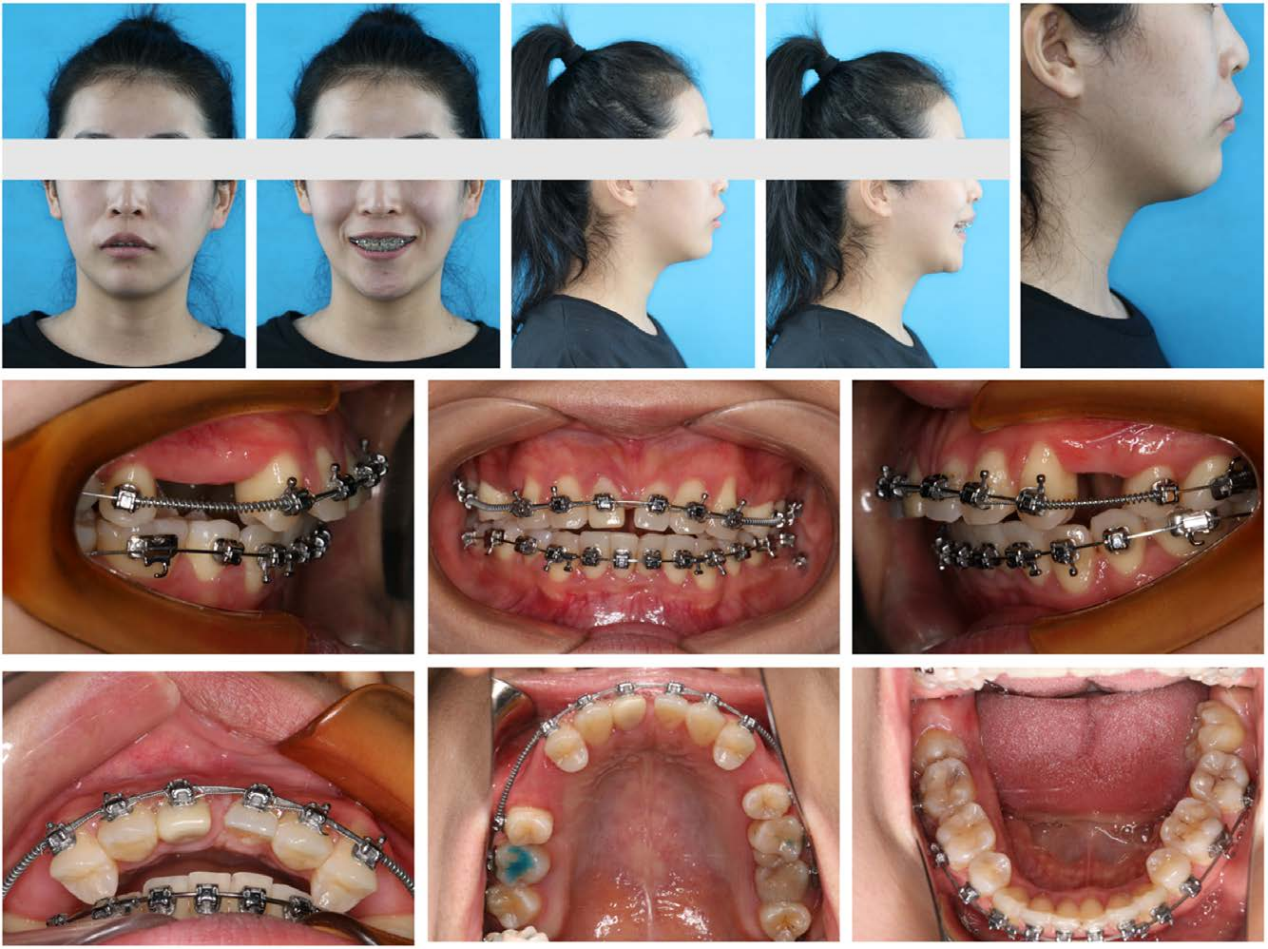
Presurgery facial and intraoral photographs.

**Figure 9.**
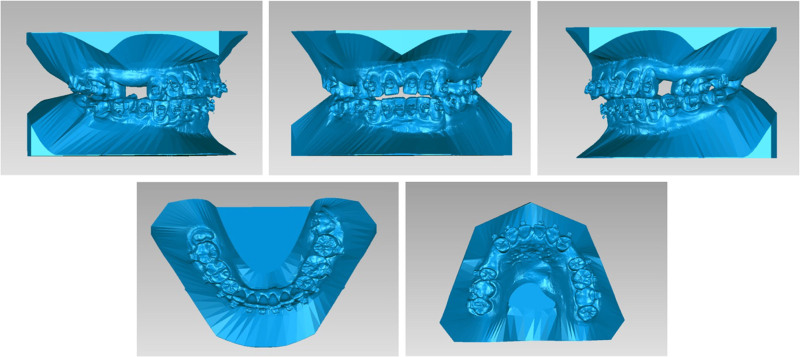
Presurgery models.

**Figure 10.**
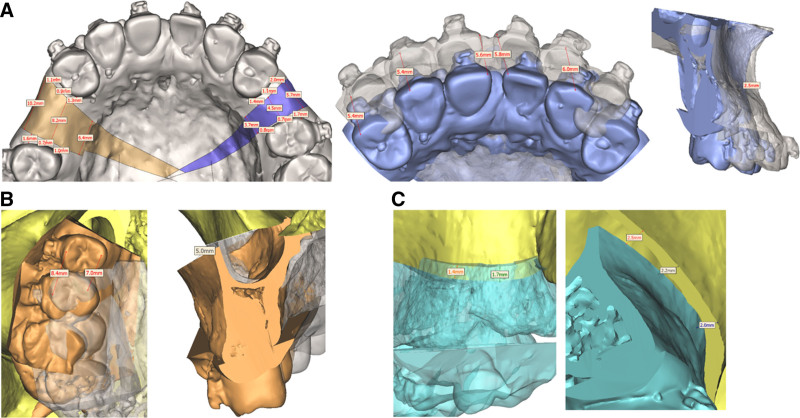
Surgical protocol design. (A) Maxillary surgery protocol, (B) upper right jaw surgery protocol, (C) upper left jaw surgery protocol.

**Figure 11.**
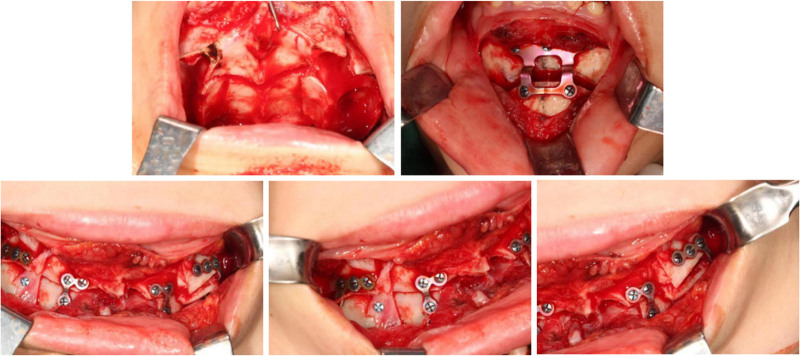
Intrasurgery photographs.

At the end of the presurgical orthodontic treatment, the upper anterior teeth were exposed to 2 mm at rest (Fig. 8). LL8 and LR8 were impacted, and there was a greater gap between the 2 maxillary premolars on the right side than on the left. The vertical opening between the anterior teeth was 1 mm and the overjet was 8 mm in the sagittal direction (Fig. 9). The surgical plan included maxillary Le Fort I osteotomy, wherein the anterior maxillary bone segment was retruded by 5 mm in the sagittal direction, vertically repositioned downward, and rotated clockwise (Fig. 10A). Additionally, the posterior segment was divided into 3 bone blocks to adjust the width. Closure of the right residual gap depends on anterior displacement of the bone mass (Figs. 10B and 11). 3D-printed occlusal plates were used to achieve precise intraoperative localization. The mandibular position was left untouched, and only the chin was water advanced 4 mm. Teeth 38 and 48 were simultaneously extracted.

### 2.6. Postsurgical orthodontics

**Figure 12.**
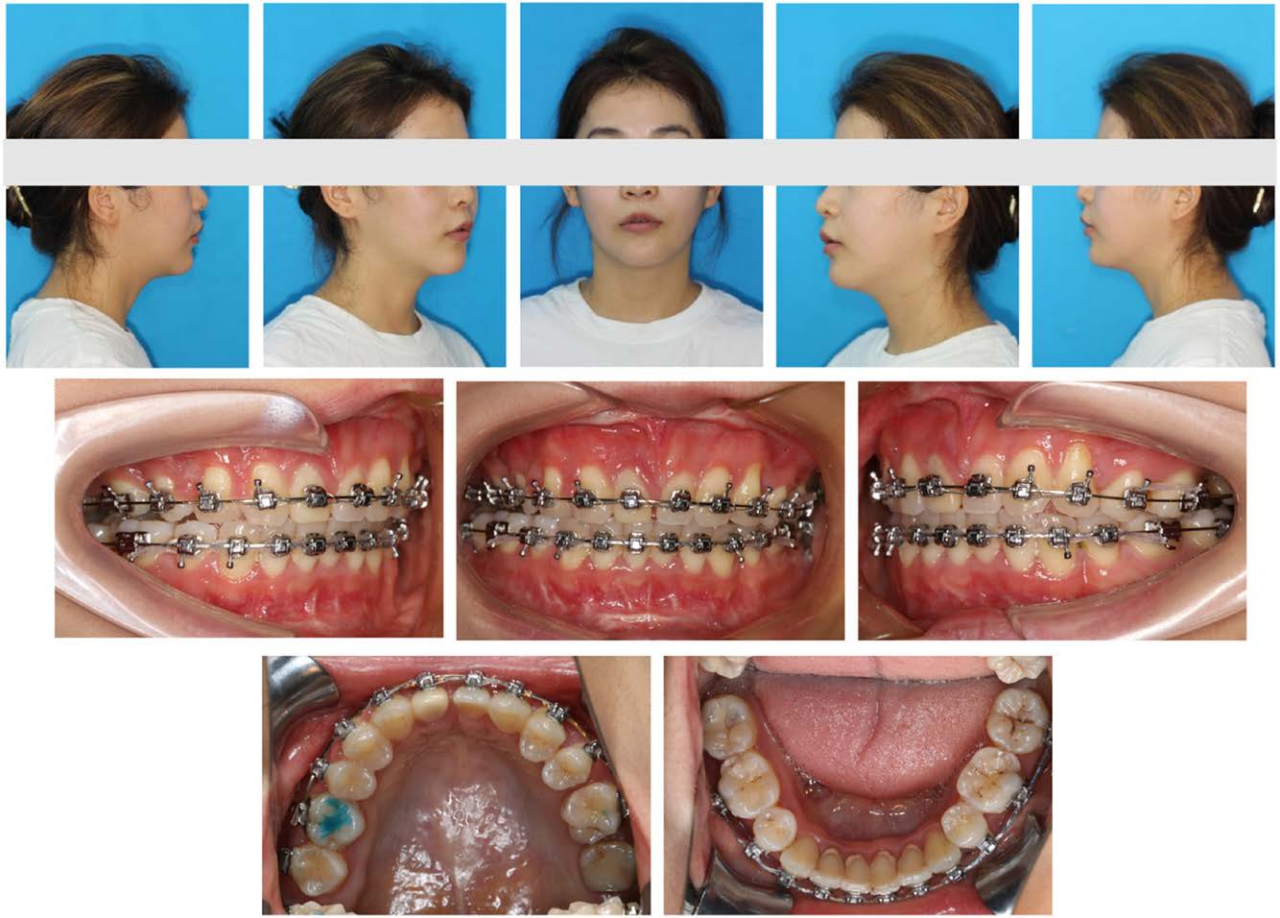
Three months postsurgery facial and intraoral photographs.

**Figure 13.**
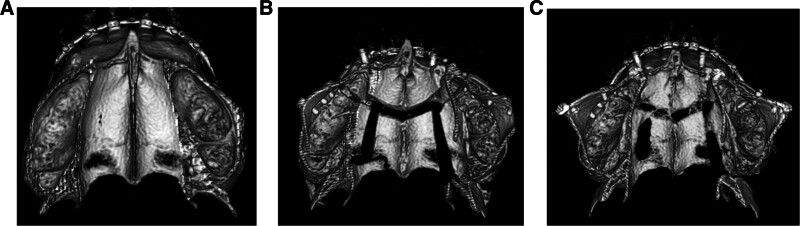
CT reconstruction at the level of palate. (A) Presurgery, (B) immediately postsurgery, (C) 3 months postsurgery.

**Figure 14.**
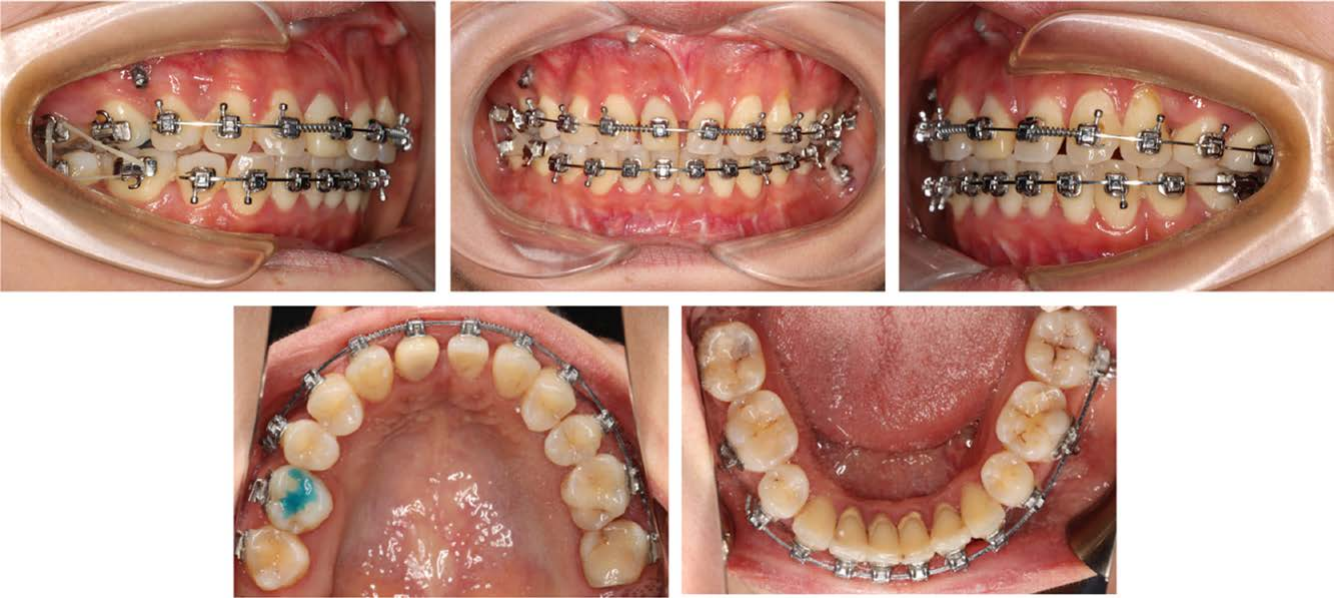
Pre-prosthodontics intraoral photographs.

The esthetic appeal of the facial shape improved using 3D-printed occlusal plates, which enabled precise intraoperative localization after orthognathic surgery, with reduced maxillary convexity and a smooth chin line (Fig. 12). Soft and hard tissues healed well, and there was significant bone regeneration at 3 months postoperatively compared with the immediate postoperative period (Fig. 13). Furthermore, the remaining gap, except for that necessary for the prosthesis, was further closed, and the occlusion was subsequently refined (Fig. 14).

### 2.7. Prosthodontics

**Figure 15.**
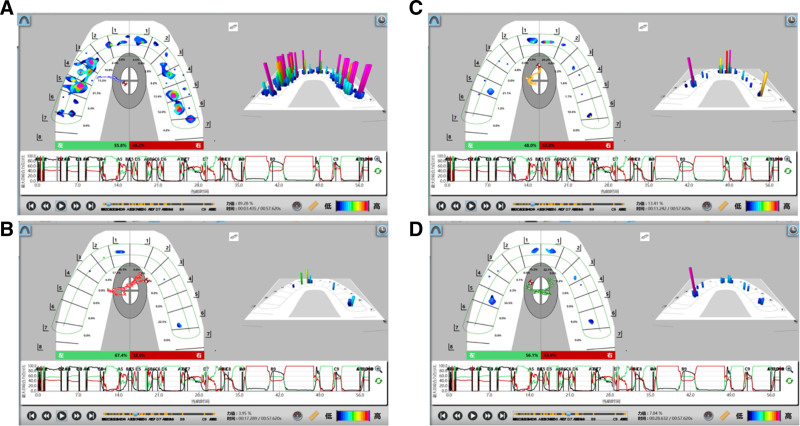
Pre-prosthodontics T scan. (A) Static occlusion, (B) anterior movement, (C) left lateral movement, (D) right lateral movement.

**Figure 16.**
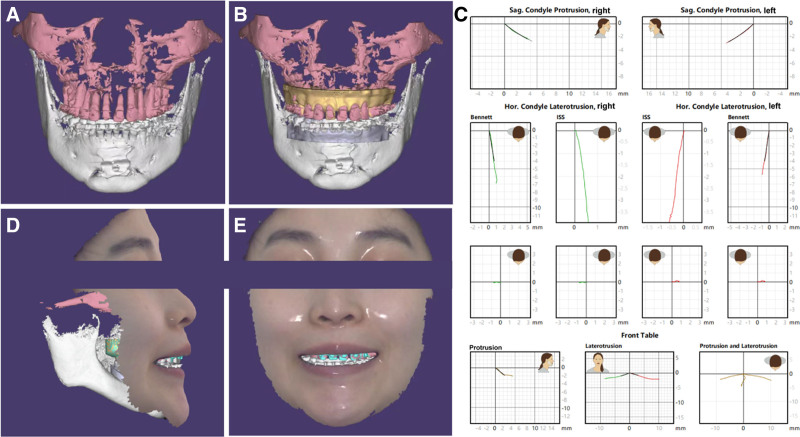
Prosthesis design. (A) CBCT, (B) intraoral scans, (C) digital cross-articulations, (D) facial scans, (E) 3D virtual patient.

**Figure 17.**
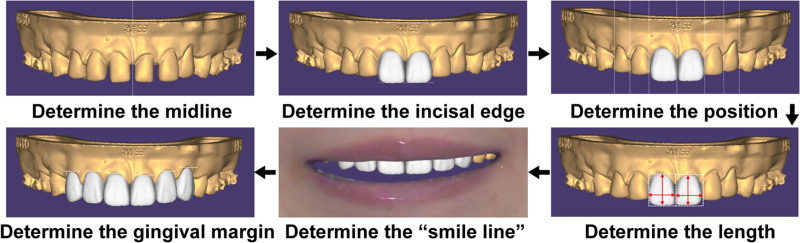
Prosthodontic treatment workflows.

In this patient, extraction of the mesial incisors led to a compromised aesthetic appearance of the anterior teeth and occlusal interference during mandibular movements (Fig. 15, Video S1, Supplemental Digital Content, http://links.lww.com/MD/O296). Hence, achieving both restoration of anterior aesthetics and establishment of normal occlusal induction are the primary objectives of prosthodontic treatment. For the esthetic and functional aspects of prosthodontic treatment involving multiple anterior teeth and to optimize minimally invasive procedures, a 3D virtual patient approach integrates facial scans, intraoral scans, and digital cross-articulations, supplanting the conventional 2D design method (Fig. 16). In the 3D virtual patient, a fully executed prosthodontic treatment of the anterior teeth displayed harmoniously coordinated hard and soft tissues in terms of aesthetics and function (Fig. 17).

### 2.8. Treatment outcomes

**Figure 18.**
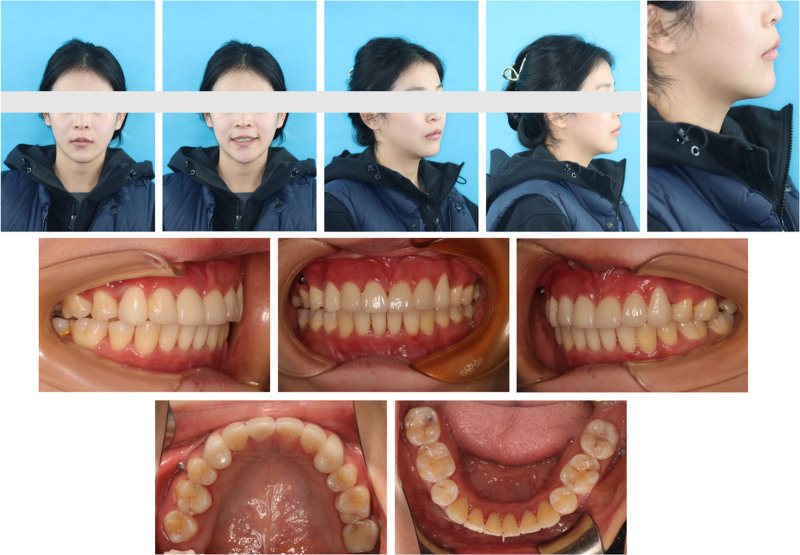
Posttreatment facial and intraoral photographs.

**Figure 19.**
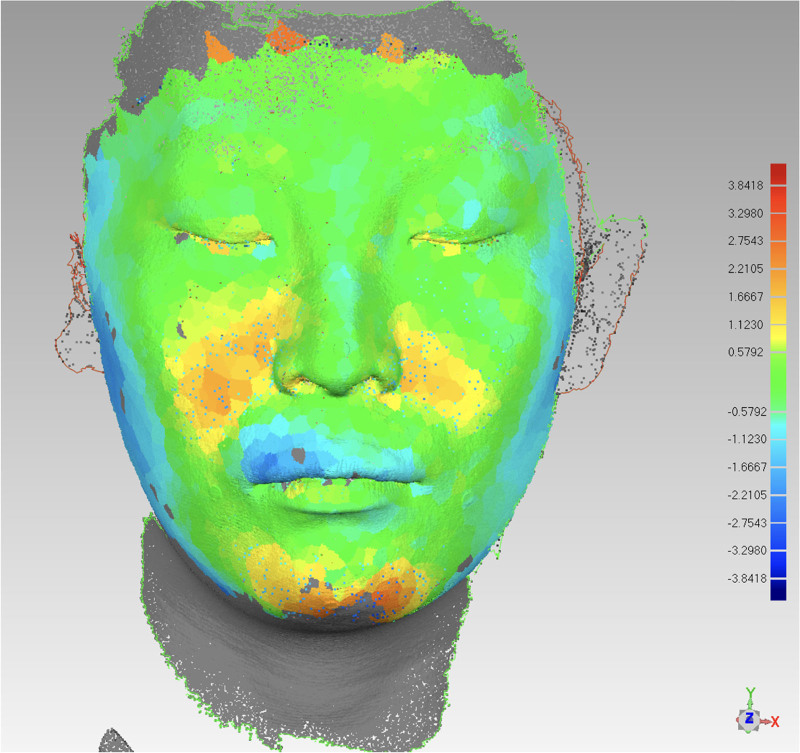
Pretreatment vs posttreatment soft tissue comparison.

**Figure 20.**
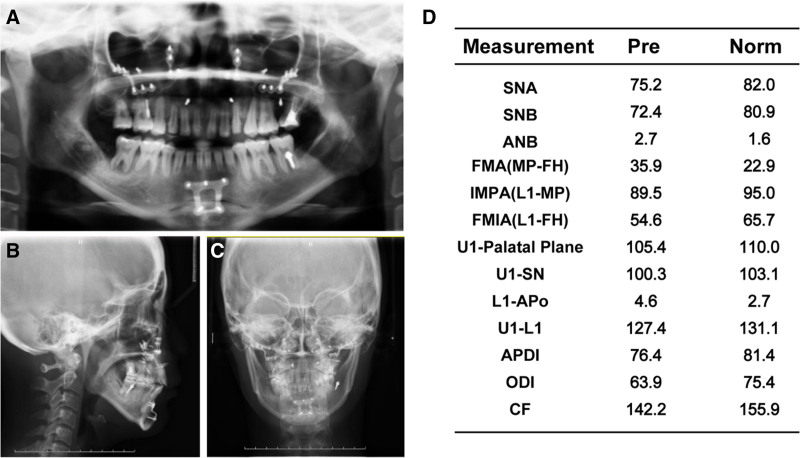
Posttreatment radiographs. (A) Lateral cephalogram. (B) Posteroanterior cephalogram. (C) Panoramic radiograph. (D) Cephalometric analysis.

**Figure 21.**
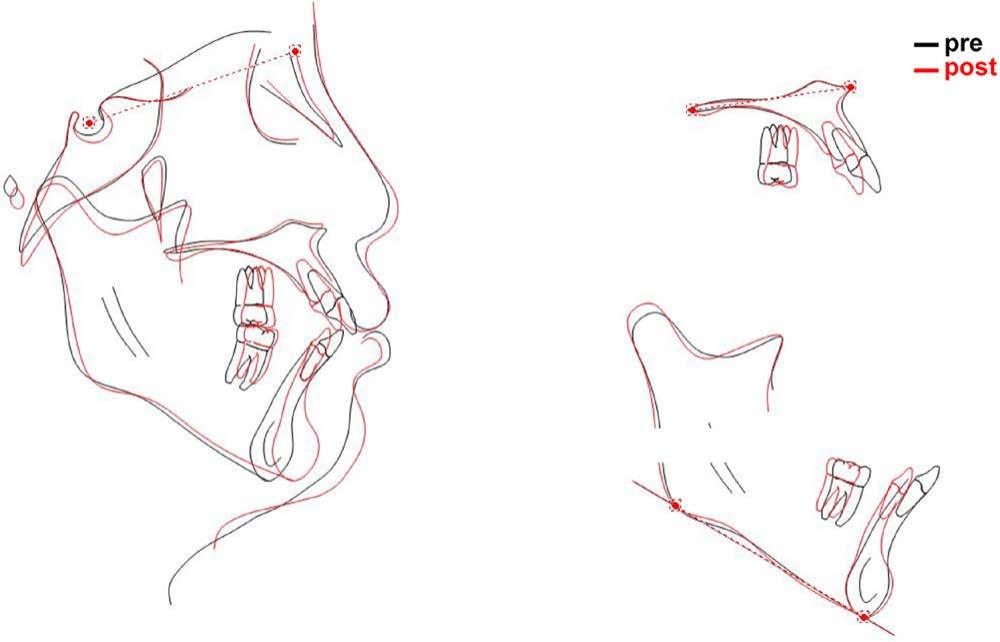
Overall cephalometric superimposition, pretreatment (black), and posttreatment (red).

**Figure 22.**
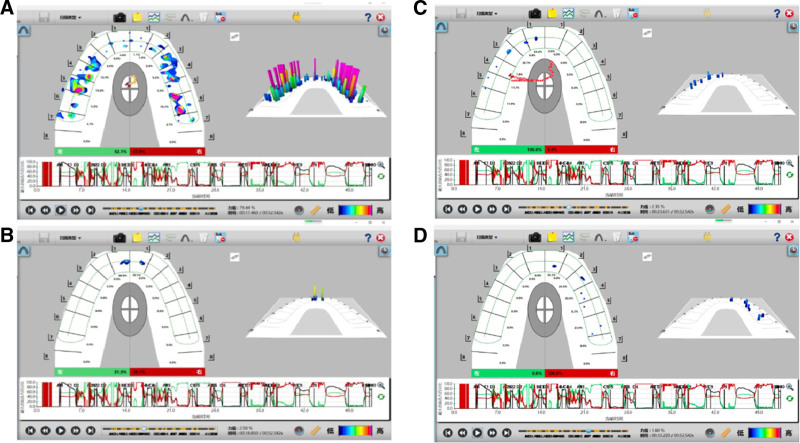
Posttreatment T scan. (A) Static occlusion, (B) anterior movement, (C) left lateral movement, (D) right lateral movement.

At the end of orthodontic treatment, the patient’s facial soft tissues were smooth, lip convexity was significantly reduced, nasolabial folds were flattened, and there was no open lip or open gingival smile (Figs. 18–21). Occlusal forces are adequate and evenly distributed, and there is no dental interference with any lateral and anterior movement (Fig. 22, Video S2, Supplemental Digital Content, http://links.lww.com/MD/O297). However, because UR7 and UR8 are lingual unicuspids, it is difficult to establish normal overbite and overjet of the right posterior teeth in the cusp-fossa interlocked condition (Fig. 18).

## 3. Discussion

In orthodontic clinical practice, an increasing number of treatment modalities have been proposed to address lateral proptosis in skeletal Class II patients. Excluding pediatric patients with developing dentition, simple orthodontic treatment with tooth extraction and combined orthodontic–orthognathic treatment emerge as the 2 primary but distinctly different approaches for adult patients. Research has confirmed that Class II patients undergoing tooth extraction can achieve effective retraction of the incisors and upper lip,^[3]^ particularly with the aid of temporary skeletal anchorage devices, which significantly enhances the efficiency of retraction.^[10]^ Therefore, for patients with mild skeletal malocclusion, tooth extraction can achieve significant aesthetic benefits with minimal economic loss and trauma. However, in patients with severe skeletal malocclusion, excessive extraction and retraction may exceed biological limits, leading to a series of adverse consequences. In such cases, combined orthodontic–orthognathic treatment becomes the optimal solution. The different skeletal patterns of Class II patients determine the selection of orthognathic surgical procedures. For example, in patients whose primary feature is maxillary protrusion, maxillary anterior osteotomy is the optimal choice,^[11]^ rather than mandibular advancement. Therefore, identifying the malocclusion patterns and severity of skeletal deformities in Class II patients is crucial for selecting an appropriate treatment plan.

In this case, the 6 elements of orofacial harmony consistently served as a guiding framework for diagnosing the patient’s malocclusion pattern and developing an effective treatment plan. These elements delineate the ideal positioning and biological constraints of teeth within the alveolar bone as well as the spatial relationships between hard and soft tissues from a three-dimensional perspective.^[1]^ This approach shifted what was previously regarded as a subjective assessment of aesthetics into an objective numerical standard. With the guidance of these 6 elements, the patient’s malocclusion pattern was identified as a maxillary protrusion accompanied by underdevelopment of the chin and excessive vertical skeletal growth. Furthermore, a comprehensive treatment plan involving combined orthodontic–orthognathic therapy has been established.

However, it is important to note that determining the target position for orthognathic surgery has consistently presented a critical challenge for practitioners during the treatment process.^[12]^ Research indicates that the correlation between soft and hard tissue displacement postsurgery varies significantly, ranging from 0.33:1 to 0.9:1.^[13]^ Therefore, relying solely on soft-tissue profiles or cephalometric data to plan the target position for orthognathic surgery is insufficient. Unlike previously reported orthognathic cases, the 6 elements were utilized in this case to determine the target position for orthognathic surgery, greatly enhancing the correlation between the hard tissues manipulated by the clinician (including the teeth and jaws) and the soft tissues that affect the aesthetic appeal.^[14,15]^ A review of the postorthognathic population indicated that the mean distance between the maxillary anterior teeth and the GALL line in women with good soft tissue aesthetics was -0.6 ± 2.1 mm,^[16]^ suggesting that satisfactory surgical outcomes align with the 6-element assessment criteria.

Appropriate occlusal guidance is crucial for oral health. Occlusal interference can affect not only the long-term stability of orthodontic treatment but also the health of the temporomandibular joint and periodontal tissues.^[17]^ However, in this case, missing incisors resulted in poor occlusal induction at the end of orthodontic treatment. The first premolar located in the position of the canine exhibits a bilobed form, which can interfere with the palatal cusp during mandibular movements. Similarly, the unilobed cusp of UR7 in the palatal aspect at UR6 also presents interference during mandibular motion. Therefore, restoring normal occlusal guidance is particularly important for orofacial region health. In this study, prosthodontic treatment was employed using 3D digital for restoration design. For maxillary lateral incisors and canines, crown restorations were used to restore anterior occlusal guidance. For the first premolars, labial veneers were used to restore lateral occlusal guidance. The results confirmed that occlusal guidance was restored after prosthodontic treatment and occlusal interferences were eliminated. The use of 3D virtual patients provides superior aesthetics and reduces the need for intraoral adjustments compared to traditional 2D methods, as it incorporates facial soft tissue references during design and better simulates the intraoral environment.^[9]^

In addition to the 3D virtual patient model utilized in prosthodontic treatment, other 3D digital technologies have been employed to improve treatment accuracy. Customized bracket base plates combined with indirect bonding systems can largely eliminate human error in bracket bonding and simplify the procedure during treatment.^[18]^ Virtual surgical planning and 3D-printed occlusal plates greatly minimize the risk of bone movement errors during surgery.^[6]^ The introduction of three-dimensional (3D) digital personalized treatment tools has simplified the treatment process and yielded precise results.

## 4. Conclusion

The design principles underlying orthodontic and orthognathic programs have been a topic of considerable debate. In this study, 6 elements of orofacial harmony were employed as a framework for program design to optimize facial aesthetics. Additionally, prosthodontic treatment contributes to enhanced anterior aesthetics and occlusal guidance, thereby promoting favorable orthodontic outcomes and maintaining oral health. Furthermore, personalized treatment modalities such as customized brackets, 3D-printed occlusal plates, and 3D virtual patient models enable significant improvements in treatment accuracy and enhance the efficiency of multidisciplinary collaboration.

## Acknowledgments

The authors thank the Department of Orthodontics, Hospital of Stomatology, Jilin University.

## Author contributions

**Conceptualization:** Mengna Duan, Guomin Wu, Yi Zhang.

**Data curation:** Huichuan Qi.

**Formal analysis:** Huichuan Qi, Zhi Mao.

**Funding acquisition:** Huichuan Qi, Yi Zhang.

**Investigation:** Zhina Wu, Xue Yang, Yi Zhang.

**Methodology:** Mengna Duan, Guomin Wu, Xue Yang.

**Project administration:** Yi Zhang.

**Resources:** Zhina Wu, Guomin Wu, Yi Zhang.

**Software:** Zhi Mao.

**Supervision:** Min Hu, Yi Zhang.

**Validation:** Min Hu, Xue Yang.

**Visualization:** Zhina Wu, Mengna Duan.

**Writing – original draft:** Jinhan Nie.

**Writing – review & editing:** Jinhan Nie.

## Supplementary Material


