# Supplementary material for: Personalized aesthetic management of skeletal Class II malocclusion with a combined approach of orthodontic, orthognathic, and prosthodontic treatment: A case report
Source: Medicine (Baltimore). 2025 Feb 7;104(6):e41326. doi: 10.1097/MD.0000000000041326 (PMC11813019; doi:10.1097/MD.0000000000041326)
Supplement: Supplementary file 1 [file medi-104-e41326-s001.docx]

**Supple Table. S1. The contents of six elements of orofacial harmony.**

| **Elements** | **Contents** | **Explanation** |
| --- | --- | --- |
| Element I | Ideal arch shape | - the roots of the teeth should align centrally within the alveolar bone under normal conditions of tooth count, adjacency, and inclination. |
| Element II | Ideal anteroposterior (AP) position of the jaws | - the center of the maxillary central incisor crown (FA point) aligns with the GALL line; - the maxillary and mandibular incisors exhibit optimal occlusal contact relationships. |
| Element III | Ideal horizontally position of the jaws | - the equality in distance between the proximal midpalatal cusps of the maxillary first molars and the central fossa of the mandibular first molars. |
| Element IV | Ideal vertical position of the jaws | - harmony among the anterior midfacial height, anterior subfacial height, and posterior height; - the FA point of the maxillary central incisor align flush with the lower edge of the upper lip; - he distance from the FA point of the lower incisor to the menton equal half the height of the lower one-third of the face; - the angle formed between the functional dental plane and the horizontal plane is less than 10°. |
| Element V | Ideal chin convexity | - the pogonion fall on the PALL line, which is the perpendicular line of the dental plane over the mandibular central incisor FA point. |
| Element VI | Ideal occlusion | - healthy, functional, and correct occlusion. |
